# Investigation of Coral-Like Cu_2_O Nano/Microstructures as Counter Electrodes for Dye-Sensitized Solar Cells

**DOI:** 10.3390/ma8095274

**Published:** 2015-08-31

**Authors:** Chih-Hung Tsai, Po-Hsi Fei, Chih-Han Chen

**Affiliations:** Department of Opto-Electronic Engineering, National Dong Hwa University, Hualien 97401, Taiwan; E-Mails: 610225011@ems.ndhu.edu.tw (P.-H.F.); 610025014@ems.ndhu.edu.tw (C.-H.C.)

**Keywords:** dye-sensitized solar cells, cuprous oxide, counter electrode, nanostructures

## Abstract

In this study, a chemical oxidation method was employed to fabricate coral-like Cu_2_O nano/microstructures on Cu foils as counter electrodes (CEs) for dye-sensitized solar cells (DSSCs). The Cu_2_O nano/microstructures were prepared at various sintering temperatures (400, 500, 600 and 700 °C) to investigate the influences of the sintering temperature on the DSSC characteristics. First, the Cu foil substrates were immersed in an aqueous solution containing (NH_4_)_2_S_2_O_8_ and NaOH. After reacting at 25 °C for 30 min, the Cu substrates were converted to Cu(OH)_2_ nanostructures. Subsequently, the nanostructures were subjected to nitrogen sintering, leading to Cu(OH)_2_ being dehydrated into CuO, which was then deoxidized to form coral-like Cu_2_O nano/microstructures. The material properties of the Cu_2_O CEs were comprehensively determined using a scanning electron microscope, energy dispersive X-ray spectrometer, X-ray diffractometer, Raman spectrometer, X-ray photoelectron spectroscope, and cyclic voltameter. The Cu_2_O CEs sintered at various temperatures were used in DSSC devices and analyzed according to the current density–voltage characteristics, incident photon-to-current conversion efficiency, and electrochemical impedance characteristics. The Cu_2_O CEs sintered at 600 °C exhibited the optimal electrode properties and DSSC performance, yielding a power conversion efficiency of 3.62%. The Cu_2_O CEs fabricated on Cu foil were generally mechanically flexible and could therefore be applied to flexible DSSCs.

## 1. Introduction

Solar cell research is receiving an increasing amount of attention from academia and various governments in response to recent discussions on energy shortages and global environmental pollution. In 1991, Grätzel *et al.* [1] used high-surface-area TiO_2_ nanoparticles to develop a novel type of solar cell called the dye-sensitized solar cell (DSSC). Compared with conventional Si-based solar cells and thin-film solar cells, DSSCs are advantageous because of their simple structure, easy fabrication process, and low cost; hence, they promptly attracted the interest of the scientific community [2,3,4,5,6,7,8,9,10]. DSSC devices comprise a transparent conductive glass substrate, TiO_2_ nanoparticle thin-film electrode, dye, electrolyte, and Pt counter electrode (CE) [11,12,13]. Over the past 20 years, numerous scientists have endeavored to enhance the efficiency of DSSCs. Such efforts have included developing technologies for fabricating porous TiO_2_ nanoparticle electrodes, designing high-performance dyes, developing solid-state electrolytes, investigating flexible substrates, and improving device encapsulation technologies. Furthermore, several studies have contributed to major breakthroughs in the field. In a typical DSSC device, CEs are a crucial component facilitating redox reactions in the electrolyte, extracting electrons from the electrolyte, and reducing the dye from an excited state to a ground state, thereby completing the dye-regeneration process [14]. The CE is usually composed of a conductive catalytic layer. The function of the conductive catalytic layer is to catalyze the reduction of the I_3_^−^ ions in the electrolyte produced during the regeneration of the oxidized dyes (through the oxidation of iodide ions in the electrolyte). The requirements for the CE in a DSSC are thus low charge-transfer resistance and high exchange current densities for effective reduction of the oxidized species, and good chemical/electrochemical stability in the electrolyte systems used in DSSCs [15]. Although Pt has traditionally been used as a CE material [16,17,18,19,20,21,22,23,24,25], its high cost increases the DSSC fabrication cost considerably. Hence, identifying a highly efficient replacement for Pt in CEs is a crucial research topic. Several previous studies have endeavored to replace Pt CEs with other more cost-effective CE materials with favorable electrochemical properties such as conductive polymers [26], carbon black [27], graphene [28], and carbon nanotubes [29].

Cu_2_O is a direct-energy gap p-type semiconductor with a band gap of approximately 2.0 eV. This compound has received considerable attention for application in photovoltaic devices because it offers several advantages such as its low cost, nontoxic nature, and abundant supply [30,31,32]. Moreover, it exhibits excellent optical absorption in the visible spectrum, can be deposited on thin films by using various coating methods, and can be fabricated using simple and economical processes. Therefore, the performance and application of solar cells can be improved by elucidating the properties of Cu_2_O. Georgieva *et al.* [33] directly electrodeposited Cu_2_O on transparent indium tin oxide (ITO) to obtain an ITO/Cu_2_O/graphite structure with a thickness of 4–6 μm and optical band gap of 2.38 eV. Jeong *et al.* [34] electrodeposited ZnO and Cu_2_O to fabricate heterojunction solar cells; however, the cells exhibited a maximal conversion efficiency of only 0.41%. Furthermore, numerous problems were unsolved, including how to achieve accurate control of the electrodeposition conditions for reducing Cu_2_O defects. Mittiga *et al.* [35] injected nitrogen and oxygen into a tube furnace to oxidize Cu and fabricate high-quality Cu_2_O; subsequently, an ion beam was employed to sputter transparent conductive oxide onto the Cu_2_O and MgF_2_ was vapor-deposited onto antireflective films. The resulting Cu_2_O/ZnO/ITO/MgF_2_ structure exhibited a conversion efficiency of 2.01%. Cu_2_O was also sputtered onto n-ZnO nanowire solar cells on ZnO–Ga/glass templates. These ZnO templates were n-type semiconductors with an energy gap of 3.37 eV. Cu_2_O is a p-type semiconductor; therefore, depositing p-Cu_2_O onto n-ZnO leads to the formation of a p–n junction [36,37]. However, previous studies have primarily adopted Cu_2_O as the working electrode (WE) in solar cells, and reports on Cu_2_O CEs in DSSCs are scarce. In the present study, we used a chemical oxidation method to fabricate coral-like Cu_2_O nano/microstructures on Cu foils for use as CEs in DSSCs. The material properties of the Cu_2_O CEs fabricated at various sintering temperatures were characterized, and subsequently, the influence of the sintering temperature on the DSSC efficiency was determined.

## 2. Experimental Section

### 2.1. Cu_2_O Counter Electrode (CE) Fabrication

**Figure 1:**
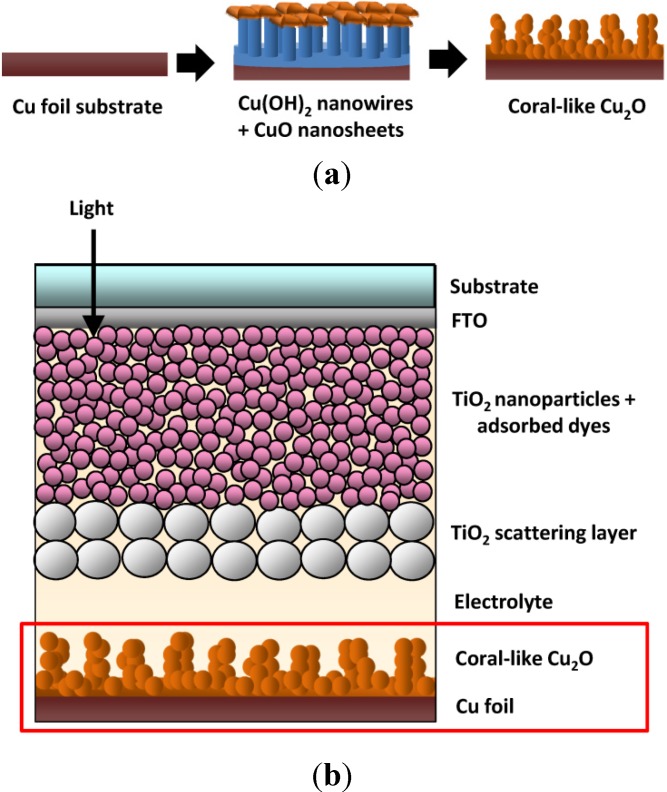
(**a**) The fabrication process of a coral-like Cu_2_O counter electrode; and (**b**) the schematic device structure of a dye-sensitized solar cells (DSSC) using the coral-like Cu_2_O counter electrode.

Figure 1a depicts the process for fabricating the Cu_2_O electrodes. The Cu foil substrates (0.5 mm thick) were chemically oxidized by being immersed in a solution containing 0.125 M (NH_4_)_2_S_2_O_8_ (Sigma-Aldrich, St. Louis, MO, USA) and 2.5 M NaOH (Sigma-Aldrich, St. Louis, MO, USA). After reacting at 25 °C for 30 min, the Cu substrates were converted to Cu(OH)_2_ nanowires. This intermediate product was sintered in a nitrogen atmosphere, leading to the dehydration of Cu(OH)_2_ and the formation of CuO nanosheets that were deoxidized into coral-like Cu_2_O nano/microstructures. For fabricating the Cu_2_O electrodes, Cu foil substrates were first ultrasonically washed in acetone, deionized water, 1 M HCl (Sigma-Aldrich, St. Louis, MO, USA), and then in deionized water again for 5 min each. Their surfaces were then blow-dried with nitrogen gas. Subsequently, 10 g of NaOH and 2.85 g of (NH_4_)_2_S_2_O_8_ were added to 10 mL of deionized water, and the mixture was ultrasonically vibrated for 30 min to evenly dissolve the powders in the aqueous solution. The Cu substrates were placed in the solution for 30 min, extracted, dried at room temperature under ambient conditions, and then placed in a tube furnace. The tube furnace was evacuated to achieve an internal pressure of 7 mTorr and subsequently purged with nitrogen gas at a rate of 300 cm^3^/min. The outlet of the nitrogen cylinder was maintained at a constant pressure, and the vacuum-purge process was performed for 3 min to ensure that the tube furnace was filled with nitrogen. Finally, the vacuum pump was shut down. When the internal nitrogen pressure reached 300 Torr, the nitrogen injection valve was closed and the Cu substrates were sintered in the nitrogen atmosphere to complete the fabrication of the Cu_2_O. The Cu substrates were sintered for 4 h at different temperatures (400, 500, 600 and 700 °C) to produce various Cu_2_O CEs for DSSCs.

### 2.2. Cu_2_O Counter Electrode (CE) Characterization

The physical, chemical, and electrochemical properties of the Cu_2_O CEs were comprehensively characterized using various analytical techniques. A scanning electron microscope (SEM) (JEOL JSM-7000F, JEOL Inc., Peabody, MA, USA) was used to observe the surface morphology of the Cu_2_O CEs. A surface profiler was used to analyze the thickness of the Cu_2_O CEs. An energy dispersive X-ray spectrometer (EDS) was employed to measure the O–Cu elemental ratios of the Cu_2_O CEs; an X-ray diffractometer (XRD) (Rigaku D/Max-2500V, Rigaku Corp., Tokyo, Japan) was used to identify the crystal structures of the Cu_2_O CEs; a Raman spectrometer was employed to verify the constituent materials of the Cu_2_O CEs; and an X-ray photoelectron spectrometer (XPS) (Thermo K-Alpha, Thermo Fisher Scientific, Waltham, MA, USA) was used to analyze the elemental and chemical composition of the Cu_2_O CE surfaces. The electrochemical activities of the Cu_2_O CEs for I_3_^−^ reduction were examined using a cyclic voltameter (CV) (CH Instruments 6116D, CH Instruments, Inc., Austin, TX, USA). The CV measurements were conducted using a three-electrode electrochemistry system. The Cu_2_O CEs under testing were used as the WE, the Pt foil was used as the CE, and Ag/Ag^+^ was used as a reference electrode. The scan rate was set at 50 mV/s and the electrolyte was an acetonitrile solution containing 10 mM LiI (Sigma-Aldrich, St. Louis, MO, USA), 1 mM I_2_ (Sigma-Aldrich, St. Louis, MO, USA), and 100 mM LiClO_4_ (Sigma-Aldrich, St. Louis, MO, USA). Furthermore, the electrochemical properties of the Cu_2_O CEs were obtained from the electrochemical impedance spectroscopy (EIS) of symmetrical sandwich device structures. Two identical Cu_2_O CEs were assembled with a sealant spacer. The electrolyte (0.6 M 1-Butyl-3-methylimidazolium iodide (BMII) (Sigma-Aldrich, St. Louis, MO, USA), 0.05 M LiI, 0.03 M I_2_, 0.5 M 4-tert-Butyloyridine (Sigma-Aldrich, St. Louis, MO, USA), 0.1 M guanidine thiocyanate (GuSCN) (Sigma-Aldrich, St. Louis, MO, USA), and a 5:1 mixture (by volume) of acetonitrile and valeronitrile (Sigma-Aldrich, St. Louis, MO, USA)) was injected between two electrodes through a drilled hole. The EIS was then conducted using an impedance analyzer (CH Instruments 6116D, CH Instruments, Inc., Austin, TX, USA), in a frequency range of 0.1 Hz to 1 MHz, under a 0 V bias and an ac amplitude of 10 mV.

### 2.3. Dye-Sensitized Solar Cells (DSSC) Fabrication

Figure 1b shows a structural diagram of a DSSC device fabricated using coral-like Cu_2_O CEs (Section 2.1 describes the Cu_2_O fabrication method). The DSSC WE was fabricated using the following method: First, a fluorine-doped tin oxide (FTO) substrate (2.2 mm thick) was cleaned and covered with 3M adhesives that were prepunched with 4-mm-diameter holes (area = approximately 0.126 cm^2^). The holes were prepunched to define the device coating area. The doctor blade method was applied to uniformly coat the area with 25-nm TiO_2_ nanoparticle paste. The coated samples were heated at 150 °C for 10 min, and then a second coating was applied to obtain 12-μm-thick TiO_2_ WEs. Subsequently, the same method was employed to coat a 200-nm TiO_2_ nanoparticle layer for use as a scattering layer. The coated samples were then sintered at 500 °C for 30 min. When cooled to 80 °C, the TiO_2_ electrodes were immersed in a dye solution for 24 h. The dye solution was composed of 0.5 mM N719 dye (Sigma-Aldrich, St. Louis, MO, USA) and 0.5 mM chenodeoxycholic acid (Sigma-Aldrich, St. Louis, MO, USA) (used as a coadsorbant) in a 1:1 volumetric mixture of acetonitrile and tert-Butyl alcohol (Sigma-Aldrich, St. Louis, MO, USA). Subsequently, 60-μm encapsulation films were cut with outer and inner dimensions of 2.5 cm × 2.5 cm and 0.8 cm × 0.8 cm, respectively, for assembling the WEs and CEs. The upper and lower electrodes were sealed by applying a constant pressure of 3 kg/cm^2^ at 130 °C for 3 min. After the electrodes had cooled, 5 μL of an electrolyte solution was injected into each device by using a micropipette. The electrolyte solution contained 0.6 M 1-Butyl-3-methylimidazolium iodide (BMII), 0.05 M LiI, 0.03 M I_2_, 0.5 M 4-tert-Butyloyridine, 0.1 M guanidine thiocyanate (GuSCN), and a 5:1 mixture (by volume) of acetonitrile and valeronitrile. The remaining 0.8 cm × 0.8 cm encapsulation films were used with glass plates to seal the CE holes by applying a pressure of 3 kg/cm^2^, to prevent the leakage and evaporation of the electrolyte. Finally, alcohol was used to clean the surface of the solar cells in preparation for experimental measurements.

### 2.4. Dye-Sensitized Solar Cells (DSSC) Characterization

The current density–voltage (J–V) characteristics of the DSSCs were measured under illumination of simulated AM 1.5G solar light from a 550-W xenon lamp solar simulator (ABET Technologies Sun 3000 Class AAA, Milford, CT, USA). To reduce the spectrum mismatch between the simulated light and AM 1.5G to below 2% in the region of 350–750 nm, the incident light intensity was calibrated to 100 mW/cm^2^ by using a reference Si photodiode equipped with an infrared-cutoff filter (KG-5, Schott, SCHOTT AG, Mainz, Germany) (the reference cell was certificated by Bunkoh-Keiki Co. Ltd., Tokyo, Japan). Photocurrent–voltage curves were obtained by applying an external bias voltage to the cell and measuring the generated photocurrent. The J–V characteristics of the DSSCs were used to determine the short-circuit current density (J_SC_), open-circuit voltage (V_OC_), fill factor (FF), and power conversion efficiency of the DSSCs. Incident monochromatic photon-to-current conversion efficiency (IPCE) spectra were measured using a 150-W xenon arc lamp (ABET Technologies, Milford, CT, USA) as the light source, which was coupled to a monochromator. The IPCE data were obtained by illuminating the solar cells with monochromatic light at a wavelength sampling interval of 5 nm between 300 and 750 nm and by measuring the short-circuit current of the solar cells. The IPCE measurements were performed under full computer control.

Electrochemical impedance spectroscopy (EIS) was employed to measure the cell impedance by using an impedance analyzer (CH Instruments 6116D, CH Instruments, Inc., Austin, TX, USA) with a frequency range of 0.1 Hz to 1 MHz. During the impedance measurements, the cell was constantly illuminated by simulated AM 1.5G solar light at an intensity of 100 mW/cm^2^. The impedance of the cell (from 0.1 Hz to 1 MHz) was measured by applying a bias at the V_OC_ of the cell (*i.e*., under no dc electric current) and by using an ac amplitude of 10 mV.

## 3. Results and Discussion

### 3.1. Cu_2_O Counter Electrodes (CE) Characterization

**Figure 2:**
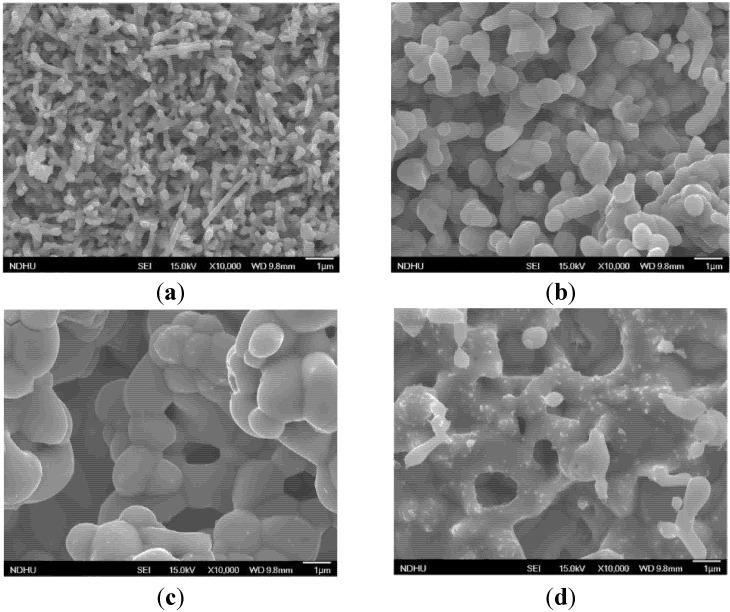
SEM images of the Cu_2_O counter electrodes sintered at (**a**) 400 °C; (**b**) 500 °C; (**c**) 600 °C; and (**d**) 700 °C.

The SEM images of Cu_2_O (Figure 2) revealed distinct morphologies among the samples fabricated at various sintering temperatures. Figure 2a shows that sintering the Cu_2_O at 400 °C caused nanowire structures to form with a diameter of 100–200 nm and length of 1–2 μm. Figure 2b shows that the Cu_2_O nanowires agglomerated into 1–2 μm coral-like structures when sintered at 500 °C. Figure 2c shows that sintering the Cu_2_O at 600 °C produced 4–5-μm coral-like structures, and Figure 2d shows that when the sintering temperature reached 700 °C, the Cu_2_O structures became integrated and several cracks developed on the surface. The thickness of the Cu_2_O CEs was measured using a surface profiler. The Cu_2_O films, which were sintered at 400, 500, 600 and 700 °C, have the thicknesses of 5.54, 5.28, 5.13 and 5.01 μm, respectively.

**Figure 3:**
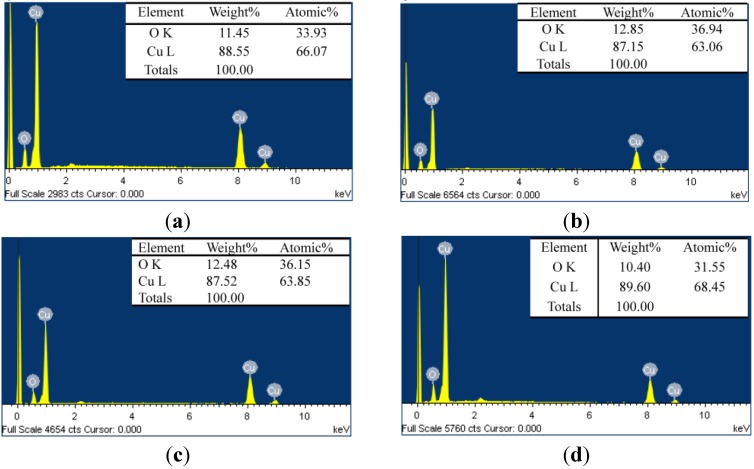
The energy-dispersive X-ray spectroscopy (EDS) analytic results of the Cu_2_O counter electrodes sintered at (**a**) 400 °C; (**b**) 500 °C; (**c**) 600 °C and (**d**) 700 °C.

Figure 3 shows the EDS results for the Cu_2_O CEs sintered at various temperatures. Figure 3a–d show that sintering the Cu_2_O at 400, 500, 600 and 700 °C yielded atomic weight (relative atomic mass) O–Cu ratios of 11.45:88.35 (33.93:66.07), 12.85:87.15 (36.94:63.06), 12.48:87.52 (36.15:63.85) and 10.40:89.60 (31.55:68.45), respectively. The EDS results show that the oxygen content of the Cu_2_O samples sintered at 500 and 600 °C was higher than that of the samples sintered at 400 °C. However, when the sintering temperature reached 700 °C, the oxygen content dropped substantially, whereas the Cu content of the Cu_2_O electrode increased. This observation was attributed to the loss of oxygen atoms in Cu_2_O at high sintering temperatures, leading to a decrease in the oxygen ratio and an increase in the Cu ratio in the Cu_2_O electrodes.

**Figure 4:**
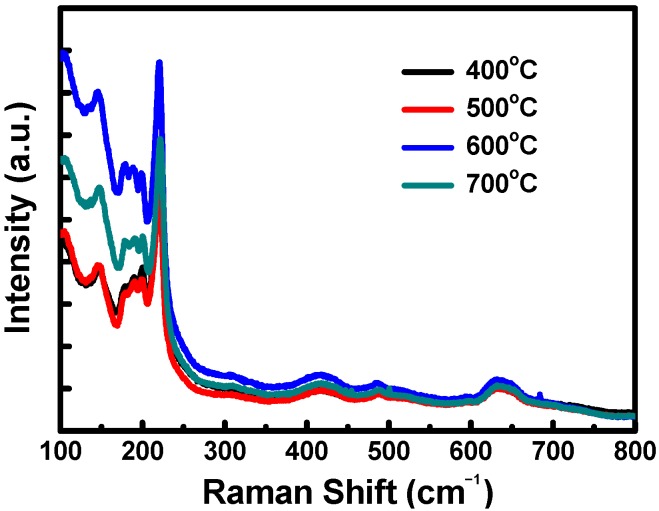
Raman spectra of the Cu_2_O counter electrodes sintered at various temperatures.

Raman spectroscopy involves using lasers to excite test samples, enabling the interaction between photons and phonons in a sample to be characterized to determine the modes of vibration and rotation of lattices and molecules. Figure 4 shows the Raman spectra of the Cu_2_O electrodes sintered at 400, 500, 600 and 700 °C in a nitrogen atmosphere. The Raman signals of Cu_2_O were observed at 151 and 220 cm^−1^; for CuO, the signals were observed at 284 and 345 cm^−1^. The Raman spectra of the Cu_2_O electrodes revealed Cu_2_O signals in the samples but no CuO signal, indicating that the electrodes sintered at 400, 500, 600 and 700 °C were converted to Cu_2_O. Additionally, the Raman spectra indicated that the Cu_2_O peak signal was most apparent in the sample sintered at 600 °C.

**Figure 5:**
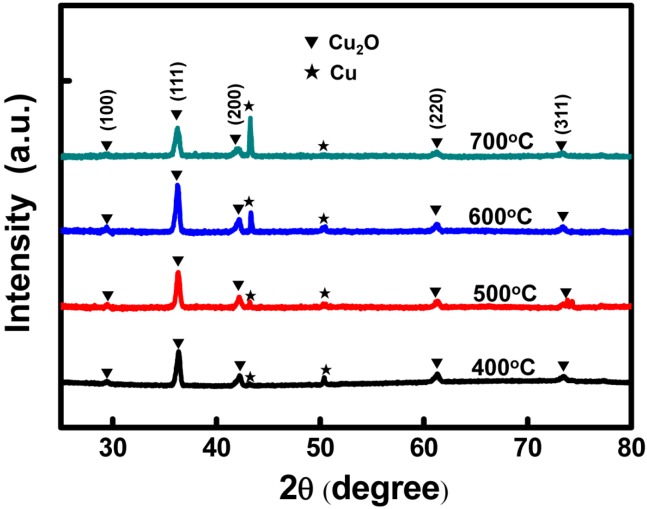
The X-ray diffraction (XRD) analytic results of the Cu_2_O counter electrodes sintered at various temperatures.

An XRD was employed to analyze the crystal structure of the Cu_2_O CEs. Figure 5 shows the XRD measurement results, in which the Cu_2_O signals correspond to the 2θ angles of 29°, 36°, 42°, 61° and 74°, which in turn correspond to the lattice planes of (110), (111), (200), (220) and (311), respectively (JCPDS card No. 78-2076). Additionally, the diffraction peaks of the Cu substrate correspond to 2θ angles of 44° and 51°. These results showed that the Cu_2_O signals were apparent in the samples after the chemical oxidation and sintering processes, thus confirming the conversion of the electrodes to Cu_2_O. For the Cu_2_O samples sintered at 600 °C, the (111) lattice plane exhibited the highest peak intensity, indicating the optimal crystal structure of the Cu_2_O. The results also showed that the Cu peak intensity at 44° increased with the sintering temperature from 400 to 700 °C, which was attributed to the gradual loss of oxygen atoms in the Cu_2_O at high sintering temperatures. The loss of oxygen atoms reduced the oxygen ratios and increased the Cu ratios of the Cu_2_O electrodes.

**Figure 6:**
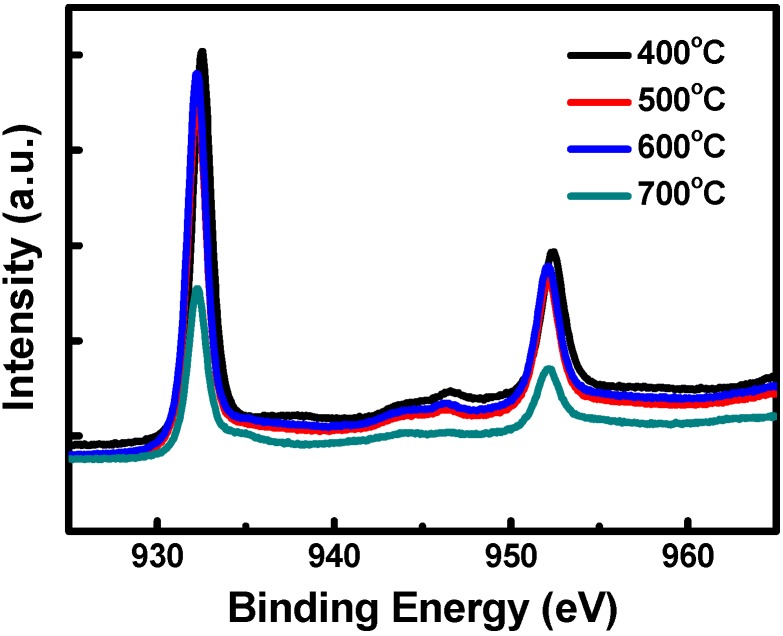
X-ray photoelectron spectroscopy (XPS) analytic results of the Cu_2_O counter electrodes sintered at various temperatures.

Figure 6 shows the XPS results for the Cu_2_O samples sintered at 400, 500, 600 and 700 °C. Because the Cu_2_O electrode substrates were Cu foils, the aqueous chemical oxidation process initially converted the Cu to Cu(OH)_2_. When the samples were sintered in a nitrogen atmosphere, the Cu(OH)_2_ was dehydrated to produce CuO, which was subsequently deoxidized to Cu_2_O. The XPS results revealed a Cu 2p_3/2_ signal at 932.2 eV and a Cu 2p_1/2_ signal at 952.1 eV, corresponding to an oxidation state of +1. The XPS spectra show that the intensities of the Cu 2p_3/2_ and Cu 2p_1/2_ signals were similar for the samples sintered at 400, 500 and 600 °C, indicating that these samples were dehydrated, deoxidized, and converted from Cu(OH)_2_ to Cu_2_O through forming the intermediate product CuO. However, for the samples sintered at 700 °C, the Cu 2p_3/2_ and Cu 2p_1/2_ signal intensities decreased, which was due to the gradual loss of oxygen atoms in the Cu_2_O at high sintering temperatures.

**Figure 7:**
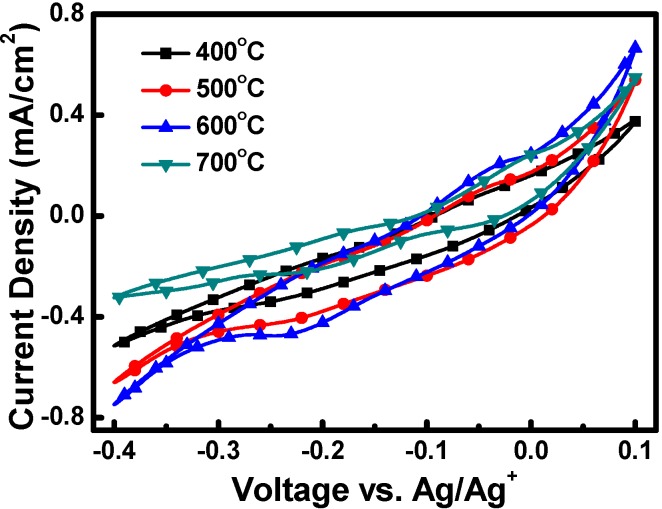
The cyclic voltammograms (CV) of the Cu_2_O counter electrodes sintered at various temperatures.

Figure 7 shows the cyclic voltammograms (CV) of the I_3_^−^/I^−^ redox couple for various Cu_2_O CEs. The results obtained with the CV showed that redox reactions occurred in the voltage ranges of −0.05 to +0.05 V (oxidation) and −0.15 to −0.25 V (reduction). In the cyclic voltammograms, the cathodic current peaks correspond to the reduction of I_3_^−^ ions through interaction with the CE. In general, the magnitude of the cathodic current peak represents the catalytic capability and/or the surface area of a CE toward reduction of I_3_^−^ in DSSCs. The cathodic current peaks of the Cu_2_O CEs were in the descending order of 600, 500, 400 and 700 °C, indicating that the catalytic capability and/or the surface area of the Cu_2_O CEs were altered by the sintering temperatures. Among the four CEs, the Cu_2_O sintered at 600 °C exhibited the largest cathodic current peak. By contrast, the Cu_2_O sintered at 400 and 700 °C revealed reduced peak current densities. In this study, the cathodic current peaks of the Cu_2_O CEs were related to both catalytic property and surface area of the Cu_2_O CEs since the Cu_2_O nano/microstructures exhibited different morphology as sintering at various temperatures.

**Figure 8:**
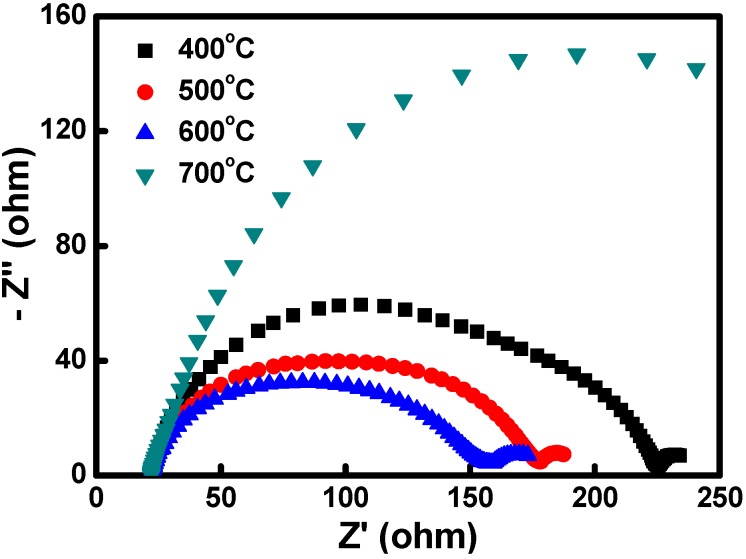
Electrochemical impedance spectra of symmetrical device structures fabricated using the Cu_2_O CEs sintered at various temperatures.

Furthermore, the electrochemical properties of the Cu_2_O CEs were obtained from the electrochemical impedance spectroscopy (EIS) of symmetrical device structures. Two identical Cu_2_O CEs were assembled with a sealant spacer, and the electrolyte was the same as used for a DSSC in this research. Figure 8 shows the impedance spectra of symmetrical cells fabricated using the Cu_2_O CEs sintered at various temperatures. The larger semicircle in the higher frequency range corresponded to the charge-transfer resistance at the CE/electrolyte interface; the smaller semicircle in the lower frequency range was associated with ion diffusion resistance in the electrolyte. Among the four Cu_2_O CEs, the Cu_2_O CEs sintered at 600 °C exhibited the smallest charge-transfer resistance at the CE/electrolyte interface. By contrast, the Cu_2_O sintered at 700 °C showed the largest charge-transfer resistance at the CE/electrolyte interface.

### 3.2. Dye-Sensitized Solar Cells (DSSC) Characterization

**Figure 9:**
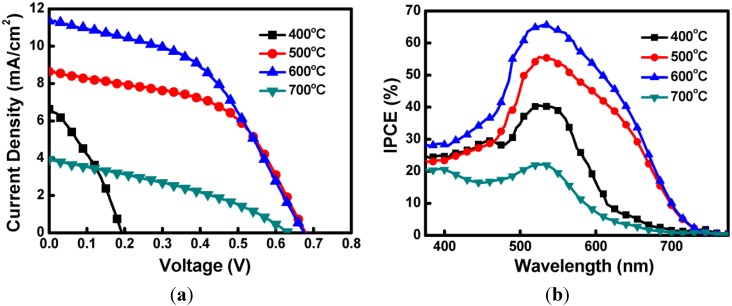
The (**a**) current density–voltage (J-V) and (**b**) incident monochromatic photon-to-current conversion efficiency (IPCE) curves of DSSCs using various Cu_2_O counter electrodes.

In this study, Cu_2_O was sintered at various temperatures to fabricate coral-like Cu_2_O CEs for DSSC devices. Figure 9a shows the J–V curves of the DSSCs fabricated using various Cu_2_O CEs. Table 1 shows the measured J–V characteristics of the DSSCs with Cu_2_O CEs sintered at 400, 500, 600 and 700 °C. Six DSSC devices were prepared and tested for each sintering condition. The deviations of the photovoltaic characteristics are also listed in Table 1. The corresponding Jsc values were 6.61, 8.64, 11.35 and 3.94 mA/cm^2^; the V_OC_ values were 0.19, 0.68, 0.68 and 0.63 V; the FF values were 0.34, 0.53, 0.47 and 0.35; and the device efficiencies were 0.43%, 3.11%, 3.62% and 0.88%. The results showed that the DSSCs with the Cu_2_O CEs sintered at 600 °C exhibited the optimal Jsc (11.35 mA/cm^2^) and power conversion efficiency (3.62%). The DSSCs with the Cu_2_O CEs sintered at 400 and 700 °C exhibited comparatively lower Jsc values and power conversion efficiencies. The J–V results were consistent with the electrode analysis and suggested that the Cu_2_O CEs fabricated at different sintering temperatures may affect the electrode properties and DSSC characteristics. Figure 9b shows the IPCE results for the DSSCs with various Cu_2_O CEs. In the figure, the IPCE wavelength peak values of the DSSCs are approximately 530 nm, corresponding to the N719 dye. The results also showed that the IPCE peak values of the DSSCs with CEs sintered at 400, 500, 600 and 700 °C are 40.5%, 55.6%, 65.6% and 22.1%, respectively. The sintering temperature for fabricating these devices can be arranged in the following descending order according to the IPCE peak values: 600, 500, 400 and 700°C. The IPCE results were consistent with the Jsc values derived from the J–V measurements.

**Figure 10:**
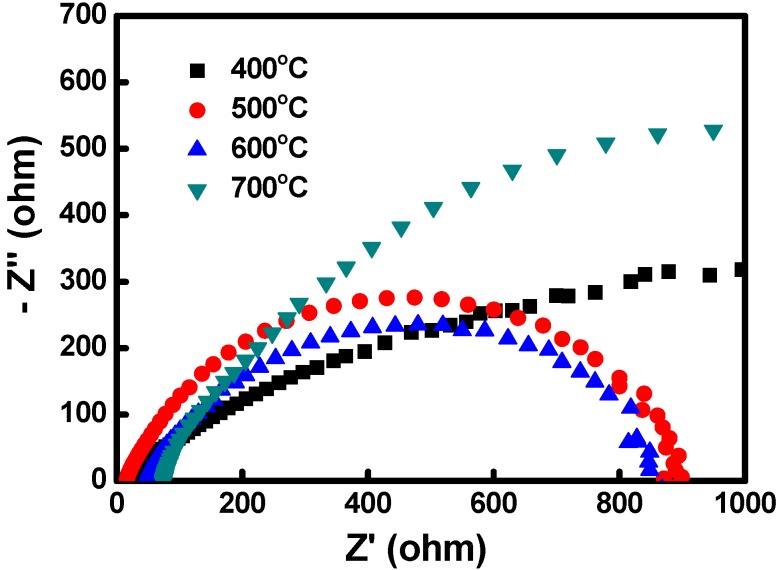
The EIS Nyquist plots of DSSCs using various Cu_2_O counter electrodes.

Figure 10 shows the EIS Nyquist plots for the various DSSC devices. EIS is a useful tool for characterizing critical interfacial charge-transfer processes in DSSCs such as the electron transfer/charge recombination at the TiO_2_/dye/electrolyte interface, electron transport in the TiO_2_ electrode, electron transfer at the CE, and I_3_^−^ transport in the electrolyte. For the investigated frequency range (0.1 Hz to 1 MHz), three regions were generally distinguished: a small semicircle in the lowest frequency range (approximately 0.1 Hz to 1 Hz), a larger semicircle in the middle frequency range (approximately 1 Hz to 1 kHz), and a smaller semicircle in the highest frequency range (>1 kHz) [38]. With the bias illumination and voltage applied, the small semicircle at the lowest frequencies was associated with ion diffusion in the electrolyte; the larger semicircle at the middle frequencies corresponded to the charge-transfer processes at the TiO_2_/dye/electrolyte interface; and the smaller semicircle at the highest frequencies corresponded to the charge-transfer processes at the CE/electrolyte interface [39]. In this study, the TiO_2_/dye/electrolyte interface impedance was considerably high; therefore, the impedances of the CE/electrolyte interface, TiO_2_/dye/electrolyte interface, and electrolyte were overlapped. The results showed that the DSSCs with the Cu_2_O CEs sintered at 600 °C exhibited the lowest impedance value (approximately 850 Ω), indicating that these CEs possessed the optimal number of photogenerated carriers; this characteristic of the CEs shows that the Cu_2_O CEs sintered at 600 °C exhibited the optimal catalytic activity and material properties. The DSSCs with the Cu_2_O sintered at 700 °C showed the highest impedance value, which resulted from the loss of oxygen atoms, inferior crystal structure, and low electrocatalytic activity of the Cu_2_O sintered at high temperatures. The EIS results were in agreement with the short-circuit currents and overall power conversion efficiencies of the DSSCs.

**Table 1:** The characteristics of device short-circuit current density (Jsc), open-circuit voltage (Voc), fill factor (FF), and device efficiency based on various Cu_2_O counter electrodes.

Cu_2_O Counter Electrode (°C)	Jsc (mA/cm^2^)	Voc (V)	Fill Factor	Efficiency (%)
400	6.61 ± 0.24	0.19 ± 0.02	0.34 ± 0.03	0.43 ± 0.10
500	8.64 ± 0.22	0.68 ± 0.01	0.53 ± 0.02	3.11 ± 0.25
600	11.35 ± 0.25	0.68 ± 0.01	0.47 ± 0.02	3.62 ± 0.31
700	3.94 ± 0.28	0.63 ± 0.02	0.35 ± 0.03	0.88 ± 0.16

## 4. Conclusions

In this study, we used a chemical oxidation method to fabricate coral-like Cu_2_O nano/microstructures on Cu foils for use as CEs in DSSCs. The Cu foil substrates were first immersed in an aqueous mixture solution containing (NH_4_)_2_S_2_O_8_ and NaOH and allowed to react at 25 °C for 30 min, which resulted in their conversion to Cu(OH)_2_ nanostructures. Through nitrogen-sintering, the Cu(OH)_2_ was dehydrated to produce CuO, which was then deoxidized to form coral-like Cu_2_O structures. The sintering processes were conducted at 400, 500, 600 and 700 °C, and the Cu_2_O CEs produced were individually characterized using SEM, EDS, XRD, Raman spectrometer, XPS, and CV to determine the influence of the sintering temperature on the material properties of the CEs. The Cu_2_O CEs were subsequently employed to fabricate DSSCs, and the properties of the DSSCs with the various Cu_2_O CEs were analyzed to determine the J–V, IPCE, and EIS characteristics. The results showed that the DSSCs with the Cu_2_O CEs sintered at 600 °C exhibited the optimal electrode properties and device efficiency (3.62%). Because Cu foil substrates are flexible, Cu_2_O CEs produced from Cu foil substrates can be employed to fabricate flexible DSSCs.
